# Characterization of the *Sideritis scardica* Extract SidTea+^TM^ and Its Effect on Physiological Profile, Metabolic Health and Redox Biomarkers in Healthy Adults: A Randomized, Double-Blind, Placebo-Controlled Study

**DOI:** 10.3390/molecules29051113

**Published:** 2024-03-01

**Authors:** Konstantinos Papanikolaou, Konstantinos Kouloridas, Anastasia Rosvoglou, Athanasios Gatsas, Kalliopi Georgakouli, Chariklia K. Deli, Dimitrios Draganidis, Aikaterini Argyropoulou, Dimitris Michailidis, Ioannis G. Fatouros, Athanasios Z. Jamurtas

**Affiliations:** 1 Department of Physical Education and Sport Science, University of Thessaly, 42150 Trikala, Greece; kpapanikolaou@uth.gr (K.P.); k.koulouridas@gmail.com (K.K.); arosvoglou@uth.gr (A.R.); t.gatsas@yahoo.gr (A.G.); delixar@uth.gr (C.K.D.); ddraganidis@uth.gr (D.D.); ifatouros@uth.gr (I.G.F.); 2 Department of Nutrition and Dietetics, University of Thessaly, 42132 Trikala, Greece; kgeorgakouli@uth.gr; 3 Department of Pharmacognosy and Natural Products Chemistry, Faculty of Pharmacy, National and Kapodistrian University of Athens, 15771 Athens, Greece; katarg@pharm.uoa.gr (A.A.); dmichail@pharm.uoa.gr (D.M.)

**Keywords:** plant extract, natural products, bioactive compounds, oxidative stress, antioxidants, metabolism, biomarkers

## Abstract

This study aimed to characterize a *Sideritis scardica* extract (SidTea+^TM^) and investigate its effect on the physiological profile, metabolic health and redox status in healthy individuals. The chemical profile and antioxidant potential of the SidTea+^TM^ extract were evaluated by UPLC-HRMS analysis and in vitro cell-free methods. Twenty-eight healthy adults participated in this randomized, double-blind, placebo-controlled study. Participants consumed 1500 mg/day of SidTea+^TM^ or a placebo for 4 weeks. At baseline and post-supplementation, participants were assessed for their anthropometric and physiological profile and provided a resting blood sample. SidTea+^TM^ decreased (*p* < 0.05) systolic blood pressure (−10.8 mmHg), mean arterial pressure (−4.5 mmHg), resting heart rate (−3.1 bpm) and handgrip strength of the non-dominant limb (−0.8 kg) whereas the placebo decreased (*p* < 0.05) handgrip strength of the dominant (−5.8 kg) and non-dominant (−3.2 kg) limb. SidTea+^TM^ also resulted in an increase (*p* < 0.05) in estimated VO_2_max (+1.1 mL/kg/min) and a reduction (*p* < 0.05) in γ-GT and SGPT enzymatic activity in serum (−3.7 and −3.3 U/L, respectively). Finally, SidTea+^TM^ increased (*p* < 0.001) total antioxidant capacity and decreased (*p* < 0.05) lipid peroxidation levels in plasma. These results indicate that SidTea+^TM^ is a potent and safe to use antioxidant that can elicit positive changes in indices of blood pressure, cardiorespiratory capacity, liver metabolism, and redox status in healthy adults over a 4-week supplementation period.

## 1. Introduction

*Sideritis* (Lamiaceae) is an endemic plant that has more than 150 species, which are mainly distributed in the Mediterranean area [1]. The main species in Greece are *S. euboea*, *S. clandestina*, *S. perfoliata L*., *S. scardica Griseb*, *S. sipylea Boiss*, *S. syriaca L*., *S. raeseri Boiss*. & *Heldr* and *S. perfoliata suscp. athoa* [2]. In the literature, extensive reference is made to the secondary metabolites of *Sideritis* species, the main ones of which are terpenoids (i.e., iridoids and kaurene species) and phenolic derivatives (i.e., flavonoids, phenolic acids and phenylethanoid glycosides) [3]. Dietary polyphenols exhibit a wide range of biological activities, such as antiatherogenic, antioxidant, anti-mutagenic, anti-inflammatory and antimicrobial properties [4,5]. Among phenolic derivatives, major significance is given to flavonoids, due to their antioxidant, anti-inflammatory, antibacterial, antiviral and anti-allergic properties in various pathologies [6,7]. Flavonoids mainly act as antioxidants, inhibiting free radical-induced cytotoxicity and lipid peroxidation and as such they can protect against chronic diseases such as atherosclerosis, cancer and others. Notably, the species *S. scardica* Griseb (synonyms: *S. florida* Boiss. & Heldr., *S. scardica* subsp. *Longibracteata,* known as mountain tea or Greek tea) [8], is a source rich in natural antioxidants and polyphenolic compounds with particularly beneficial effects for human health [9,10]. Concerning its geographic distribution, *S. scardica* can be found at Central Balkan Peninsula, namely in the southwest regions of Albania, northeastern regions of Greece, central and western regions of North Macedonia, the southern regions of Bulgaria, and the European part of Turkey. Specifically, these compounds have been shown to inhibit free radical-induced cytotoxicity and lipid peroxidation, acting as agents of inhibition of cell proliferation through the inhibition of tumor growth and as weak agonists or antagonists of estrogens through the regulation of endogenous hormonal activity [11]. To this extent, they can protect against chronic and neurodegenerative diseases such as atherosclerosis, cancer and Alzheimer’s disease [12,13,14].

For many years, there has been a worldwide shift toward the use of natural products, particularly in the fields of medicine, cosmetics and food [15,16]. In corroboration, the European Medicines Agency (EMA) accepts the use of certain herbal drugs as key ingredients for the development of new treatments as well as traditional medicines whose safety is confirmed by prolonged safe use over a period of years [17]. At the same time, EMA strongly urges the strengthening of research activity in order to develop new products that will have potential industrial use at the level of pharmaceutical products, food supplements and even functional food products [17]. *S. scardica* plant is said to derive its name from its ability to heal wounds from iron objects, or by others from the tooth-shaped calyx of its flower resembling a spear point. Dioscorides described both the botanical characteristics and the medicinal uses of *Sideritis* in antiquity in his work De Materia Medica in 50 AD [2]. In modern Greece, infusions from *Sideritis* plant have a pleasant aroma with a bitter taste and continue to be popular and widespread drinks for the traditional treatment of common colds, fever and gastric disorders. At some regions infusions from *S. scardica* are traditionally used as anti-inflammatory and anti-rheumatic. Moreover, preparations of *S. scardica* are used for the treatment of chest illnesses, bronchitis, intestinal diseases, pulmonary emphysema, angina pectoris and urinary tract infections [9]. Additionally, the protection against oxidative damage is one of the most widely described attributes of polyphenols of *S. scardica* and is connected with their antioxidant activity [9,18]. In recent human clinical studies, *S. scardica* supplementation resulted in improved cognitive performance due to increased cerebral blood flow [19], while *S. euboea* extract supplementation was effective in lowering total cholesterol levels in adults [20]. Nevertheless, controversial data also exist in the literature, with some studies failing to report the beneficial effects of *Sideritis* extract supplementation on antioxidant status and parameters of cardiometabolic health in healthy subjects [20,21]. However, to date, there is no information regarding the effects of *S. scardica* extract supplementation on the physiological characteristics, metabolic health, oxidative damage and redox status regulation in the adult population.

Therefore, the purpose of this study was to characterize the chemical profile and antioxidant capacity of a novel *S. scardica* plant extract (SidTea+^TM^) originating from the Greek mountain Taygetos and to investigate its effect on the physiological profile, metabolic health and redox status biomarkers in apparently healthy individuals over a 4-week supplementation period.

## 2. Results

### 2.1. SidTea+^TM^ Extract Characterization

**Figure 1:**
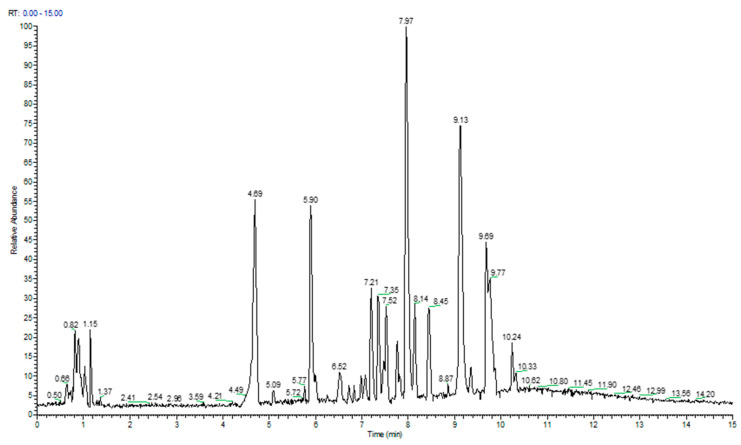
UPLC-ESD-HRMS chromatogram of SidTea+^TM^ extract analysis.

The UPLC-HRMS analysis revealed that the chemical profile of SidTea+^TM^ extract was characterized by a plethora of metabolites. Specifically, more than 35 metabolites were identified, among other compounds, phenolic acids, phenylethanoid glycosides and flavonoid glucosides were detected (Table 1). The compounds 3, 4, 22, 28 and 31 were the most abundant, as shown in the UPLC-HRMS chromatogram, (Figure 1). The TPC was estimated to be 112 mg GA/g extract, while the DDPH assay showed that the antioxidant potential of the extract was promising with an IC_50_ of 157.82 μg/mL.

### 2.2. Dietary Nutrient Intake Analysis

The dietary nutrient intake analysis showed that there were no differences in the assessed nutrients between the two groups either before or following the SidTea+^TM^ and placebo supplementation period, indicating that participants followed the same dietary habits during the 4-week supplementation period. Table 2 shows the analysis of the dietary records provided by the participants before and following supplementation.

### 2.3. Response of Anthropometric, Physiological and Performance Markers

The response of anthropometric, physiological and performance variables after SidTea+^TM^ and placebo supplementation is shown in Table 3. SidTea+^TM^ supplementation reduced systolic blood pressure by 10.9 mmHg (*p* = 0.002; ES: 0.84; 95%CI: 0.07/1.61), mean arterial pressure by 4.5 mmHg (*p* = 0.026; ES: 0.53; 95%CI: −0.23/1.28) and resting heart rate by 3.1 bpm (*p* = 0.036; ES: 0.26; 95%CI: −0.48/1.01). Both SidTea+^TM^ and placebo supplementation resulted in a decrease in handgrip strength in the non-dominant limb (SidTea+^TM^: −2.8 kg; *p* = 0.013; ES: 0.32; 95%CI: −0.43/1.06 and placebo: −3.4 kg; *p* = 0.034; ES: 0.30; 95%CI: −0.44/1.05), while there was a significant decrease (−5.8 kg) in handgrip strength of the dominant limb only in the placebo group (*p* < 0.001; ES: 0.47; 95%CI: −0.28/1.22). SidTea+^TM^ supplementation also resulted in an increase (*p* = 0.031; ES: −0.16; 95%CI: −0.90/0.59) in estimated VO_2_max by 1.1 mL/kg/min. There were no significant differences after SidTea+^TM^ or placebo supplementation on body mass, BMI, hip and waist circumferences, fat percentage, fat and lean body mass, and diastolic blood pressure.

### 2.4. Response of Complete Blood Count Markers

The response of the complete blood count markers is shown in Table 4. Both SidTea+^TM^ and placebo supplementation did not change the parameters examined by the complete blood count analysis (*p* > 0.05).

### 2.5. Response of Metabolic Markers

Table 5 shows the responses of metabolic markers following SidTea+^TM^ and placebo supplementation. Following the administration of SidTea+^TM^ there was an 8.5% decrease in the levels of γ-GT (*p* = 0.039; ES: 0.24; 95%CI: −0.51/0.98) and a 9.7% decrease in the levels of SGPT (*p* = 0.049; ES: 0.43; 95%CI: −0.32/1.18) in serum. Glucose, total cholesterol, HDL, LDL, triglycerides, bilirubin, creatinine, uric acid, SGOT and LDH did not show any change following the supplementation period in both groups (*p* > 0.05).

### 2.6. Response of Redox Biomarkers

**Figure 2:**
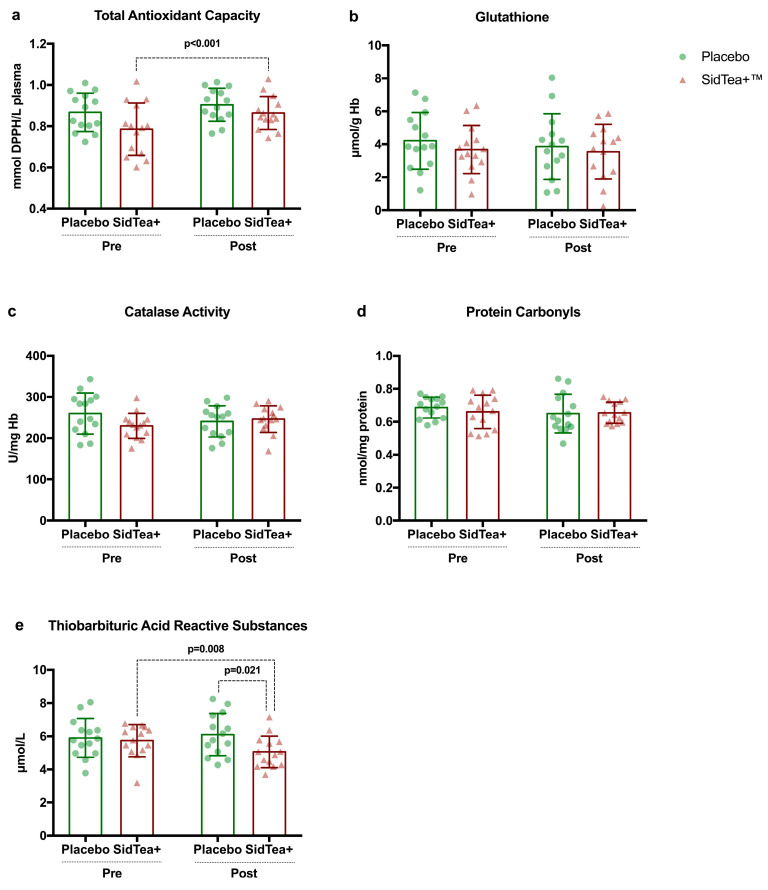
(**a**) Total antioxidant capacity—TAC; (**b**) glutathione; (**c**) catalase activity; (**d**) protein carbonyls; (**e**) thiobarbituric acid reactive substances—TBARS at baseline and following SidTea+^TM^ and placebo supplementation.

Figure 2 shows the response of redox biomarkers following SidTea+^TM^ and placebo supplementation. SidTea+^TM^ supplementation resulted in an increase (*p* < 0.001; ES: −0.71; 95%CI: −1.48/0.005) in plasma TAC and a reduction (*p* = 0.008; ES: 0.68; 95%CI: −0.08/1.44) in levels of plasma TBARS. Additionally, following SidTea+^TM^ supplementation, levels of plasma TBARS were significantly lower (*p* = 0.021; ES: −0.90; 95%CI: −1.68/−0.12) compared to the placebo group. No differences were reported in glutathione, catalase activity, and protein carbonyls levels following SidTea+^TM^ and placebo supplementation (*p* > 0.05).

## 3. Discussion

Our results indicate that SidTea+^TM^ contains high amounts of phenolic acids, phenylethanoid glycosides and flavonoid glucosides with a promising antioxidant radical scavenging capacity. Furthermore, a 4-week supplementation period with SidTea+^TM^ decreased systolic blood pressure and mean arterial pressure, resting heart rate, and strength of the non-dominant arm, and it increased estimated VO_2_max. Additionally, SidTea+^TM^ supplementation resulted in a significant decrease in indices related to liver function (γ-GT and SGPT enzyme activity) and positive changes in redox biomarkers (increase in TAC and decrease in TBARS). Percentage wise, from the variables examined and related to physiological characteristics, there was a positive change in 3 of the 13 variables (23%) and in terms of performance in one of the 3 variables (33%). Overall, 2 out of 12 variables (17%) related to metabolism showed a positive response, while 2 out of 5 (40%) variables examining oxidative stress showed a positive response.

The UPLC-HRMS analysis showed that the SidTea+^TM^ chemical profile is characterized by a wide variety of compounds. Among these compounds, SidTea+^TM^ was found to be rich in phenolic acids (e.g., chlorogenic acid), phenylethanoid and iridoid glycosides (e.g., melittoside derivative) and flavonoid glucosides (e.g., apigenin). The above-mentioned results come to total agreement with previous bibliographic reports on the chemical characteristics of *Sideritis* species [22,23]. Regarding the properties of these chemical compounds, in vitro and in vivo studies have confirmed the role of chlorogenic acid in controlling oxidative stress and inflammation by exerting antioxidant and DNA-protective effects [24]. Additionally, apigenin flavonoid, which was also found in abundance in SidTea+^TM^ extract, has been shown to exhibit antioxidative and radical scavenging activities, probably through its interaction with redox-sensitive molecular signaling pathways such as NF-kB, Nrf2, MAPK, and P13/Akt [25,26]. Based on the TPC and DPPH scavenging activity results, SidTea+^TM^ extract showed a promising antioxidant potential, an observation that is in line with other studies of the *Sideritis* species [23,27,28].

The results of the present study showed a significant drop in systolic arterial pressure of approximately 10 mmHg. Recent data indicate that a 5 mmHg drop in systolic blood pressure is associated with a 10% reduction in the risk of a cardiovascular event [29]. Additionally, SidTea+^TM^ supplementation resulted in a significant drop in mean arterial pressure of approximately 5 mmHg. Mean arterial pressure is the determining factor of the pressure of perfusion received by the various organs of the body. An increase in mean arterial pressure levels above 90 mmHg progressively increases the risk of cardiovascular diseases, such as stroke, and mortality [30]. The observed effects on blood pressure following SidTea+^TM^ supplementation appear to be similar to other plant-derived extracts in regulating blood pressure in normotensive human subjects. Specifically, supplementation with *Crocus sativus*, *Nigella sativa* oil and *Panax ginseng* extracts have resulted in reductions in systolic blood pressure of ~5–11 mmHg and mean arterial pressure of ~10 mmHg in healthy humans [31,32]. SidTea+^TM^ supplementation also significantly reduced resting heart rate by 3.1 bpm and increased estimated VO_2_max level by 1.1 mL/kg/min. There is strong evidence to suggest that resting heart rate and VO_2_max can be used as independent predictors of atherosclerosis and other cardiovascular diseases [33,34,35]. Possible mechanisms that could lead to positive cardiovascular effects due to SidTea+^TM^ extract supplementation are mainly attributed to endothelium-mediated vasodilation effects through the induction of the endothelial nitric oxide synthase (eNOS) pathway. eNOS signaling has a protective function for the cardiovascular system via the production of nitric oxide (NO) which acts as an antioxidant [36] and vasodilator [37] in the endothelium and the vascular smooth muscle cells and regulates vascular tone and blood pressure. Importantly, the functional loss of NO production is involved in the pathogenesis of hypertension and results in endothelial-dependent vasodilation impairment which precedes the structural changes (i.e., arterial stiffness, plague deposition) in the vasculature and it has been characterized as the earliest event in the development of hypertension even in apparently healthy individuals [37,38]. *S. scardica* extracts contain compounds like flavonoids and polyphenols that have been shown to exert strong antioxidant and vasodilation properties on blood vessels [19,39] and therefore could lead to a decrease in blood pressure due to their interaction with NO synthesis and availability. Indeed, data suggest that polyphenols like ferulic acid can positively modulate NO synthase expression [40], while chlorogenic acid can induce the generation of NO from nitrite and induce smooth muscle cells relaxation [41]. Interestingly, in normotensive individuals with normal blood pressure mechanisms, NO has been identified as an important regulator of systemic vascular resistance and blood pressure further corroborating its regulatory role in both physiological and pathological states [42].

Furthermore, recent results indicate that supplementation with a *S. scardica* extract (950 mg) resulted in improved cerebral blood flow, as indicated through near-infrared spectroscopy, and a significant reduction in anxiety state [19], providing further explanation for the reduction in blood pressure and heart rate seen in our study. In addition, the phenolic hydroxyl group in the structure of polyphenols can scavenge free radicals and inhibit lipid peroxidation of the endothelium leading to a vasodilation effect that will cause decreases in arterial pressure. TBARS, an index of lipid peroxidation, decreased following SidTea+^TM^ supplementation by ~14%, whereas TAC increased by ~13%, indicating that supplementation with a *S. scardica* extract can positively affect redox status biomarkers, and this effect could possibly have contributed to the decrease in blood pressure and heart rate observed in the present study. In corroboration, increased antioxidant activity from *S. scardica* extract supplementation has been also presented by previous research [43]. However, it should be noted that the observed effects on blood pressure seen in our study may have been influenced by an acute vasodilatory effect between the last dose of the SidTea+^TM^ supplement and the measurement of blood pressure (~8 h period). To this extent, more clinical data are needed to draw concrete conclusions on the chronic blood pressure-lowering effects of SidTea+^TM^ extract proposed herein. 

SidTea+^TM^ as well as placebo supplementation resulted in a significant decrease in handgrip strength of the non-dominant limb but the reduction in handgrip strength in the dominant limb was only evident after placebo supplementation. Handgrip strength plays a significant role in daily living and is an indicator of an individual’s ability to perform motor tasks. Moreover, handgrip strength is related to conditions like frailty and sarcopenia [44,45] and there are a variety of factors, like aging, sedentary lifestyle, injury and lack of sleep, that can contribute to a decrease in handgrip strength [46]. Since participants’ lifestyle conditions (i.e., nutrition and physical activity) did not change during the experimental period, we could assume that the only possible reason for explaining the decrease in handgrip strength are upper limb disuse and the effect of aging, especially in the older individuals of the study. Undoubtedly, this is an observation that needs further research in order to elucidate the mechanisms for this handgrip strength decline.

SidTea+^TM^ supplementation did not show any significant changes in the indicators evaluated with the complete blood count analysis. Notably, we did not find any human studies assessing the effects of *S. scardica* supplementation on parameters of complete blood count. In addition, no changes in glucose, total cholesterol, HDL, LDL, and triglyceride levels were found among markers related to lipidemic profile. These results are partly verified in a study by Kassi et al. [20] which showed that *S. euboea* extract supplementation (another *Sideritis* species) reduced total cholesterol levels but failed to elicit any other effect on the lipidemic and inflammatory status of humans. In another study, *Nitaria retusa* extract did not affect the lipidemic profile of healthy participants but induced a significant reduction in triglycerides levels and an increase in HDL cholesterol in the obese participants [47]. In the present study, no significant changes were detected in the levels of creatinine, uric acid, bilirubin, lactate dehydrogenase and SGOT. However, two markers related to liver function (i.e., γ-GT and SGPT) showed a significant decrease, following SidTea+^TM^ supplementation. Plant-derived flavonoids have been shown to regulate several processes related to liver metabolism and function such as enzymatic activity, cytokine production and apoptosis and to protect against liver injury [48]. Previous research using *S. scardica* infusion did not report beneficial effects on liver enzymatic function in humans [49]. However, the aforementioned study used only two cups (8oz) of herbal infusion and lasted only 6 days, whereas our study used a higher dose (1500 mg) of *S. scardica* extract and lasted 28 days. Moreover, other studies on other plant-derived extracts such as *Nitaria retusa* and green tea extract supplementation failed to positively affect liver function in healthy subjects [47,50], corroborating the superior efficiency of SidTea+^TM^ extract in regulating liver function in apparently healthy individuals. Nonetheless, it should be noted that different extracts, doses and consumption duration could be determining factors for the different results observed between the studies.

Five redox biomarkers were examined, and two of them showed a significant change. More specifically, SidTea+^TM^ supplementation increased plasma TAC by 11.6%, a biomarker that shows how efficiently plasma antioxidants can neutralize free radicals produced, and reduced plasma TBARS by 10.8%, an indicator of lipid peroxidation. Numerous in vitro and in vivo studies have shown that plant-derived extracts and their phytoconstituents can protect cells and tissues form oxidative damage through direct radical-scavenging mechanisms but also through indirect modulation of redox-sensitive pathways and the induction of cellular antioxidant responses [51]. Recent research indicates that *S. scardica* is rich in phenolics and flavonoids and can exert significant antioxidant activity [3,18]. *Sideritis* extracts have been shown to produce beneficial effects on characteristics of dementia and Alzheimer’s disease since they can inhibit the aggregation and toxicity of amyloid-β in vivo [13,52]. However, there are no clinical studies assessing the impact of *S. scardica* extract on redox status. The only available clinical study that assessed the antioxidant effects of *S. euboea* extract did not report significant difference in total antioxidant status of the serum following a 1-month supplementation period [20]. Differences in the content and concentration of phenolic derivatives and flavonoids between the two *Sideritis* extracts (*scardica* vs. *euboea*) as well as the supplementation scheme applied could explain the different results between the two studies and the high efficiency of SidTea+^TM^ extract in upregulating antioxidant status in healthy humans. Regarding the effects of other plant-derived extracts in humans, green tea extract supplementation failed to decrease plasma F_2_-isoprostanes concentration in adult women [53], while grape pomace extract consumption (along with a high-fat meal) was effective in reducing postprandial levels of TBARS by ~10–15% in normal-weight women [54]. Future clinical studies on *S. scardica* extract supplementation should investigate the safety, effects and mechanisms of action of different doses (titration studies) and supplementation duration (>4 weeks) on indices of metabolism, inflammation, redox status and physical performance in different age groups and pathophysiological conditions (e.g., aging and obesity) in order to elucidate the health-promoting effects of these extracts.

In conclusion, the results of the present study indicate that SidTea+^TM^ *S. scardica* extract supplement is a potent and safe-to-use antioxidant that can induce positive effects on cardiovascular health (blood pressure, resting heart rate and cardiorespiratory capacity), liver metabolism and redox status in healthy adults over a 4-week supplementation period.

## 4. Materials and Methods

### 4.1. Experimental Design

**Figure 3:**
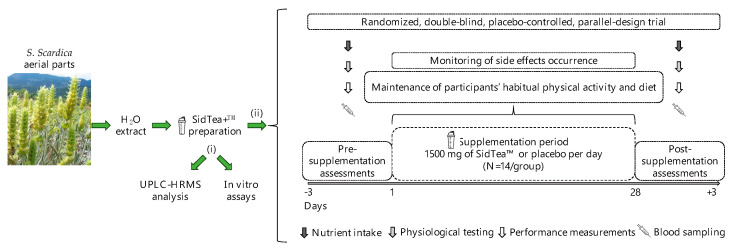
The experimental design of the study.

**Figure 4:**
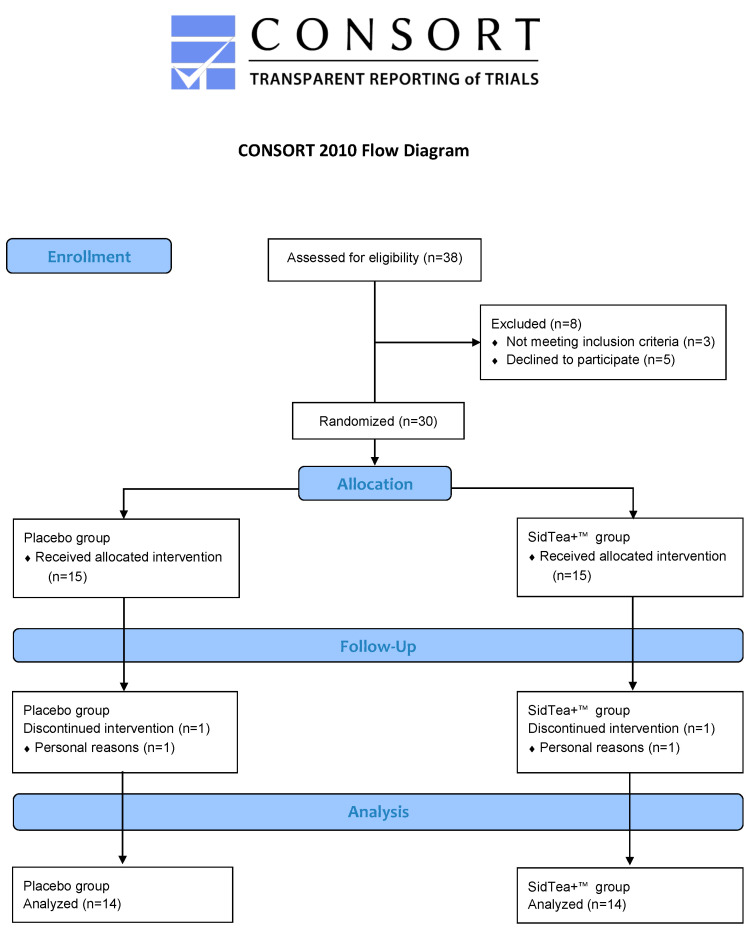
The CONSORT flow diagram of the study.

For the SidTea+^TM^ extract production, *S. scardica* aerial parts were collected from local producers in the Greek mountain region of Taygetos, and the plant extract was prepared following standardized procedures. Following SidTea+^TM^ production, the chemical profile of the extract was analyzed by ultra-performance liquid chromatography coupled with high-resolution mass spectrometry (UPLC-HRMS). The Total Phenolic Content (TPC) and the DPPH radical scavenging capacity of the extract were also determined by in vitro cell-free methods. A preliminary power analysis [55] based on the design of the study and adjusted for a medium effect size and within–between interactions indicated a total sample size of 28 participants (N = 14 per group), for a power of 80%. Initially, 30 healthy adults (15 men and 15 women) participated in this randomized, double-blind, placebo-controlled, parallel design study, but after randomization, 2 participants dropped out due to personal reasons. Exclusion criteria for the study included the following: (a) presence of cardiovascular, liver, renal, hematological or endocrine diseases, (b) musculoskeletal injury, (c) consumption of vitamin, mineral or dietary supplements for more than 3 months before as well as during the study, and (d) allergies. Participants were randomly allocated by a researcher not involved in data collection and analysis using the random table method to either a SidTea+^TM^ or a placebo supplementation group (SidTea+^TM^: 6 men, 8 women, 39.5 ± 13.9 years; placebo: 8 men, 6 women, 42.1 ± 11.6 years) and they consumed 1500 mg/day of SidTea+^TM^ or placebo, distributed in three equal doses (every 8 h) for 4 weeks. Both the participants and researchers assessing outcomes were blinded to the supplementation group. After enrollment, participants were instructed to continue their habitual physical activity and diet, maintaining the same levels of consumption of fruits, vegetables and plants, and avoiding the consumption of flavonoids or herbal beverages during the study period. Participants were instructed by a registered dietician (author K.G.) on how to record their diet for 3 days before the study and asked to follow the same dietary pattern for 3 days before the post-supplementation assessments. During the pre-supplementation assessments, participants visited the laboratory two times. During their first visit, anthropometric (body mass, body height, body mass index, body fat, waist, and hip circumferences) and physiological measurements (resting heart rate and blood pressure) were performed. During their second visit to the laboratory, the first blood sample was collected and participants had their handgrip strength and VO_2_max (indirect estimation) assessed. Participants were then given the exact amount of pre-packed SidTea+^TM^ or placebo (according to the allocation sequence), which they were instructed to consume daily for the following 4 weeks. At the end of the 4-week supplementation period, participants returned to the laboratory and underwent the same assessments (in the same order) as the pre-supplementation period. Figure 3 shows the experimental design of the study, Figure 4 depicts the CONSORT flow diagram and Table S1 contains the CONSORT checklist of the information reported in the present randomized trial.

### 4.2. SidTea+^TM^ and Placebo Production

For the production of SidTea+^TM^, *S. scardica* aerial parts were purchased from local producers in the Greek mountain region of Taygetos. Taking into consideration the polymorphism, the ecotype variation, and the frequent hybridization of genus Sideritis species, the initial plant material was selected from supervised cultivations from producers at specific location with specific reproducing methods. The plant was grounded into a fine homogeneous powder and macerated in a pilot scale tank. More specifically, H_2_O was used as an extraction solvent with a plant/solvent ratio of 1:15 (*w*/*v*). The extraction time was 3 h at 85 °C, with stirring set at 50 rpm. The final extract was successively filtrated from 250 μm, 100 μm, 50 μm, 25 μm and 10 μm. A percolator was used to decrease the solvent volume and to reach the total solids (TS) of the extract at 5%. Afterwards, maltodextrin (D.E. 12) was added to the solution in a TS/maltodextrin ratio of 8:2 (*w*/*w*). For the final formulation of the extract, a pilot scale spray dryer was used with inlet and outlet temperatures set at 170 °C and 80 °C, respectively. Gelatin capsules were filled with the SidTea+^TM^ extract or placebo (maltodextrin) and were identical in appearance and taste. Maltodextrin, solvents, chemicals and reagents were purchased from Merck (Athens, Greece).

### 4.3. UPLC-HRMS Analysis

For the chemical characterization of SidTea+^TM^, a UPLC-HRMS analysis was employed in an H-class Acquity UPLC system (Waters, Milford, MA, USA) coupled to an LTQ-Orbitrap XL hybrid mass spectrometer (Thermo Scientific, Waltham, MA, USA). The used column was a Fortis C-18 (1.7 µm, 150 × 2.1 mm). The sample was prepared in the final concentration of 500 μg/mL, diluted in 1:1 MeOH:H_2_O. The elution system consisted of water acidified with 0.1% formic acid (A) and acetonitrile (B) in the following gradient mode: 0–2 min 2% B, 2 to 18 min from 2% to 100% B, 18 to 20 min 100% B, 20–21 min from 100% to 2% B, and 21 to 25 min 2% B. The flow rate was set at 0.4 mL/min, and the injection volume was 10 µL. Ionization was achieved in negative ion mode (ESI−) at 350 °C. The mass spectrometric parameters were sheath gas and aux gas flow rate of 40 and 10 units, respectively; capillary voltage, 30 V; and tube lens, 100 V for the positive mode and capillary voltage of −20 V and tube lens of −80 V for the negative mode. The mass range was adjusted from 115 to 1000 *m*/*z*.

### 4.4. In Vitro Cell-Free Assays

The Total Phenolic Content (TPC) of SidTea+^TM^ was determined by employing the Folin–Ciocalteu method, according to a reported methodology, with minor modifications [28]. Folin–Ciocalteu solution was prepared with 10% dilution in distilled water and 7.5% sodium carbonate. In 96-well plates, 25 μL of extract was dissolved in DMSO, 125 μL Folin–Ciocalteu solution and 100 μL Na_2_CO_3_ solution were mixed. The plates were incubated for 30 min in the dark. A microplate reader (Infinite 200 PRO, TECAN, Mannedorf, Switzerland) was used for the measurement of sample absorbance at 765 nm. The results were expressed as milligrams of gallic acid equivalent (GAE) per gram of extract, based on the reference gallic acid calibration curve (at a linearity range of 1–10 μg/mL with the equation y = 0.083x + 0.046, R2 = 0.998).

For the determination of the radical scavenging activity of SidTea+^TM^ against stable DPPH, a spectrometric method was employed as described previously [28]. Specifically, 100 μL of the sample solution (200 mg/L), diluted in DMSO, were added to 1.9 mL of a 315 μM DPPH ethanolic solution and allowed to react for 30 min at 37 °C. A blank sample was prepared by adding 100 μL of DMSO to the DPPH solution. Subsequently, the absorbance was measured at 515 nm, and the % scavenging was calculated using the following equation, where A0 is the blank absorbance and A is the sample absorbance: % Scavenging = ((*A*0 − *A*)/*A*0) × 100%.

### 4.5. SidTea+^TM^ and Placebo Supplementation

Participants orally consumed 1500 mg/day of SidTea+^TM^ or placebo, distributed in 3 equal doses (every 8 h) for a total duration of 4 weeks. Due to lack of robust clinical data on the physiological dose of *S. scardica* dry extracts in humans (excluding herbal infusions), SidTea+^TM^ extract supplementation dose was calculated based on the available EMA monograph on Herbal Medicinal Products for comminuted herbal infusions [17] and supplementation duration was adopted from previous clinical studies of Sideritis extract supplementation in humans [19,20]. Based on our extraction procedure, plant/solvent ratio, TS concentration and supplementation scheme, the amount of *S. scardica* dry extract preparation (SidTea+^TM^) used in the present study was within the recommended dose of comminuted *S. scardica* herbal tea infusion for adults, as proposed by EMA [17]. The daily distribution of SidTea+^TM^ supplement in 3 equal doses (500 mg each), every 8 h, was implemented in order to minimize the incidence of potential side effects. Participants received the capsules pre-packed by the same researcher responsible for group stratification. Participants’ adherence to the supplementation scheme was verified by frequent (every 3 days) mobile phone notifications and contacts with a research assistant. SidTea+^TM^ and placebo supplements were well tolerated by the participants as no side effects were reported throughout the experimental period.

### 4.6. Dietary Nutrient Intake Analysis

Participants were instructed by a registered dietician on how to complete a 3-day diet record (2 mid-week days and 1 weekend day) and record food and liquid portions and size. Colored food images were also provided and participants were asked to describe their food intake in as much detail as possible [56]. Dietary records were analyzed using the ScienceFit Diet 200A software (Science Technologies, Athens, Greece) and the average intake of the following dietary nutrients was calculated: Energy (kcal/day), carbohydrates (g/day and % of total energy/day), fat (g/day and % of total energy/day), protein (g/day and % of total energy/day), cholesterol (mg/day), selenium (μg/day), vitamin A (IU/day), vitamin C (mg/day), vitamin E (mg/day).

### 4.7. Anthropometric, Physiological and Performance Measurements

Participants were lightly dressed and barefoot and the standing height was measured to the nearest 0.5 cm (Stadiometer 208; Seca, Birmingham, UK) and body mass to the nearest 0.05 kg (Beam Balance 710; Seca, Birmingham, UK). Body mass index (BMI) was calculated using the following equation: BMI = body mass/height^2^. Body fat percentage (% fat) was measured with a Tanita Body Fat Monitor/Scale TBF-521 (Tanita, Inc., Arlington Heights, IL, USA). Waist and hip circumferences were obtained with a measuring tape. Waist-to-hip ratio (WHR) was calculated using the following equation: WHR = waist circumference/hip circumference. Resting heart rate was recorded using telemetry via a heart rate monitor (Polar Tester S610TM, Electro Oy, Kempele, Finland), with subjects resting in a sitting position for 15 min prior to taking a resting heart rate value. Systolic and diastolic blood pressure was measured with participants in a sitting position using a manual sphygmomanometer (FC-101 Aneroid Sphygmomanometer; Focal Corporation, Kashiwa, Japan). Subjects rested in a sitting position for 15 min prior to taking resting blood pressure values. Mean arterial pressure was calculated using the following equation: Mean arterial pressure = [systolic blood pressure + (2 × diastolic blood pressure)]/3 [57]. A hand dynamometer (Jamar, 5030J1, Jamar Technologies^TM^, Horsham, PA, USA) was used to assess hand grip strength with procedures described previously [58]. Briefly, subjects performed a standard warm-up that included three preliminary trials in order to be familiarized with the test procedure. The testing protocol consisted of three maximal attempts for each hand with 1 min rest between trials. The highest recorded value of the three attempts was used for the analysis. The test–retest reliability for the assessment of maximal handgrip strength in untrained adults is ICC > 0.90 [58]. Maximal oxygen consumption (VO_2_max) was indirectly estimated through a single-stage treadmill test with procedures described in detail previously [59]. During VO_2_max testing, heart rate was recorded through telemetry by a heart rate monitor.

### 4.8. Blood Sampling

Blood samples (12 mL) were drawn from a forearm vein following an overnight fast. For serum separation, a blood sample was transferred into a separate tube containing clot activator, left at room temperature for 20 min, and then centrifuged at 1370× *g* for 10 min at 4 °C in order to obtain the supernatant (serum) that was aliquoted into Eppendorf^TM^ tubes and stored at −80 °C for later determination of metabolic markers. For plasma and erythrocyte lysate separation, a blood portion was collected to a tube containing ethylenediaminetetraacetic (EDTA) acid anticoagulant and centrifuged at 1370× *g* for 10 min at 4 °C. Plasma was collected into Eppendorf^TM^ tubes and stored at −80 °C for later analysis of redox status biomarkers. After plasma extraction, an equal volume of distilled water was added to the tube containing packed erythrocytes, the sample was vigorously mixed and then centrifuged at 4000× *g* for 15 min at 4 °C. The supernatant (erythrocyte lysate) was then aliquoted into Eppendorf^TM^ tubes and stored at −80 °C for later determination of hemoglobin (Hb), and redox status biomarkers. A blood portion (~2 mL) was collected into pre-filled EDTA tubes and used to determine the complete blood count parameters measured by an automatic hematology analyzer (Mythic 18 Orphee, Orphee Medical, Geneva, Switzerland).

### 4.9. Metabolic and Redox Assays

Determination of glucose, total cholesterol, triglycerides, high-density lipoprotein (HDL), bilirubin, uric acid, creatinine, gamma-glutamyl transferase (γ-GT), serum glutamic-oxaloacetic transaminase (SGOT), serum glutamate pyruvate transaminase (SGPT), lactate dehydrogenase (LDH) and total protein was performed in an automated clinical chemistry analyzer (HumaStar 200, Human diagnostics, Zafeiropoulos, Greece) using commercially available kits (Human diagnostics, Zafeiropoulos, Greece) and as described previously [60]. Low-density lipoprotein (LDL) was calculated by the following: equation LDL cholesterol = total cholesterol − HDL cholesterol − (triglycerides/5) [57]. Redox biomarkers and hemoglobin concentration were analyzed on a Hitachi 2001 UV/VIS spectrophotometer (Hitachi Instruments Inc., San Jose, CA, USA) as previously described [61]. Plasma samples were analyzed for total antioxidant capacity (TAC), thiobarbituric acid reactive substances (TBARS) and protein carbonyls (PC). PC concentration was normalized to the total protein concentration of the plasma. Erythrocyte lysate samples were analyzed for glutathione (GSH), catalase activity and Hb. GSH and catalase activity were normalized to Hb concentration. Each sample underwent only one freeze–thaw cycle and each marker was analyzed in duplicates on the same day.

### 4.10. Statistical Analyses

The normality of the data distribution was examined with the Shapiro–Wilk test. A two-way ANOVA (group × time) was applied to examine possible differences in the parameters tested. When a significant interaction was observed, a Bonferroni correction with pairwise comparisons was applied. Effect sizes with corresponding 95% confidence intervals were calculated according to Hedges’ method corrected for bias and were interpreted as minimal, small-, medium- and large-sized for values 0.1–0.2, 0.2–0.5, 0.5–0.8 and >0.8, respectively, according to Cohen’s d criteria. Statistical significance was set at *p* < 0.05. Statistical analyses were performed with IBM SPSS Statistics, version 29.0 (SPSS Inc., Chicago, IL, USA). The results are presented as mean ± SD.

## Figures and Tables

**Table 1:** Chromatographic and spectrometric characteristics of compounds identified in the SidTea+^TM^ extract using UPLC-ESI(-)-HRMS.

No.	Compounds	Rt (min)	Molecular Formula	[M-H]− (*m*/*z*)	Δm (ppm)	RDBeq
1.	Quinic acid	0.89	C_7_H_12_O_6_	191.0562	0.46	2.5
2.	Malic acid	1.02	C_4_H_6_O_5_	133.0145	2.13	2.5
3.	Melittoside derivative	4.69	C_22_H_34_O_17_	569.1723	0.07	15.5
4.	Chlorogenic acid	5.90	C_16_H_18_O_9_	353.0870	−2.28	8.5
5.	Neochlorogenic acid	6.03	C_16_H_18_O_9_	353.0868	−2.87	8.5
6.	Apigenin 7-O-allosyl(1 → 2)-glucoside	6.25	C_27_H_30_O_15_	593.1512	0.19	13.5
7.	Feruloylquinic acid	6.77	C_17_H_20_O_9_	367.1037	0.41	8.5
8.	Echinacoside	7.00	C_35_H_46_O_20_	785.2510	−0.14	13.5
9.	Lavandulifolioside	7.08	C_34_H_44_O_19_	755.2393	1.29	13.5
10.	Verbascoside	7.21	C_29_H_36_O_15_	623.1981	0.75	12.5
11.	Forsythoside B	7.35	C_34_H_44_O_19_	755.2382	−2.69	13.5
12.	Isoscutellarein 7-O-allosyl(1 → 2)-glucoside	7.35	C_27_H_30_O_16_	609.1450	1.47	13.5
13.	Luteolin 7-O-allosyl-(1 → 2)-[6″-O-acetyl]-glucoside	7.45	C_29_H_32_O_17_	651.1550	−2.61	14.5
14.	Isoverbascoside	7.46	C_29_H_36_O_15_	623.1965	−2.56	12.5
15.	Hypolaetin 7-O-[6‴-O-acetyl]-allosyl(1 → 2)-glucoside	7.53	C_29_H_32_O_18_	667.1516	0.68	14.5
16.	3′-O-Methylhypolaetin 7-O-allosyl(1 → 2)-glucoside	7.57	C_28_H_32_O_17_	639.1567	0.77	13.5
17.	Allysonoside (Isomer I)	7.57	C_35_H_46_O_19_	769.2561	0.30	13.5
18.	Leucosceptoside A	7.73	C_30_H_38_O_15_	637.2138	0.40	12.5
19.	Apigenin 7-O-glucoside	7.78	C_21_H_20_O_10_	431.0984	0.82	12.5
20.	Isoscutellarein 7-O-[6‴-O-acetyl]-allosyl(1 → 2)-glucoside	7.81	C_29_H_32_O_17_	651.1549	−3.09	14.5
21.	Apigenin 7-O-[6‴-O-acetyl]-allosyl(1 → 2)-glucoside	7.90	C_29_H_32_O_16_	635.1598	−3.11	14.5
22.	Isoscutellarein 7-O-allosyl-(1 → 2)-[6″-O-acetyl]-glucoside	7.97	C_29_H_32_O_17_	651.1551	−2.31	14.5
23.	3′-O-Methylhypolaetin 7-O-[6‴-O-acetyl] -allosyl(1 → 2)-glucoside	8.14	C_30_H_34_O_18_	681.1659	−1.98	14.5
24.	4′-O-Methylisoscutellarein 7-O-allosyl(1→2)glucoside	8.45	C_28_H_32_O_16_	623.1594	−3.75	13.5
25.	4′-O-Methylhypolaetin 7-O-[6‴-O-acetyl] -allosyl(1 → 2)-glucoside	8.56	C_30_H_34_O_18_	681.1652	−2.96	14.5
26.	Hypolaetin 7-O-[6‴-O-acetyl] -allosyl-(1 → 2) [6″-O-acetyl]-glucoside	8.62	C_31_H_34_O_19_	709.1602	−3.01	15.5
27.	Isoscutellarein 7-O-[6‴-O-acetyl]-allosyl(1 → 2)-[6″-O-acetyl]-glucoside	9.08	C_31_H_34_O_18_	693.1661	−1.67	15.5
28.	4′-O-Methylisoscutellarein 7-O-[6‴-O-acetyl]-allosyl(1 → 2)-glucoside	9.13	C_30_H_34_O_17_	665.1704	−2.87	14.5
29.	3′-O-Methylhypolaetin 7-O-[6‴-O-acetyl]-allosyl-(1 → 2)-[6″-O-acetyl]-glucoside	9.23	C_32_H_36_O_19_	723.1756	−3.05	15.5
30.	Apigenin 7-(6″-p-coumaroylglucoside)	9.35	C_30_H_26_O_12_	577.1339	−2.15	18.5
31.	Apigenin 7-(4″-p-coumaroylglucoside)	9.69	C_30_H_26_O_12_	577.1339	−2.25	18.5
32.	Apigenin	9.72	C_15_H_10_O_5_	269.0451	−1.66	11.5
33.	4′-O-Methylisoscutellarein 7-O-[6‴-O-acetyl]-allosyl-(1 → 2)-[6″-O-acetyl]-glucoside	10.24	C_32_H_36_O_18_	707.1808	−2.97	15.5
34.	Luteolin derivative	10.97	C_17_H_14_O_6_	313.0710	−2.40	11.5
35.	4′-O-Methylhypolaetin 7-O-[6‴-O-acetyl] -allosyl-(1 → 2) [6″-O-acetyl]-glucoside	11.12	C_32_H_36_O_19_	723.1765	−1.87	15.5
36.	Hypolaetin trimethylether (Isomer I)	11.46	C_18_H_16_O_7_	343.0826	0.71	11.5
37.	Hypolaetin trimethylether (Isomer II)	11.58	C_18_H_16_O_7_	343.0828	1.29	11.5

**Table 2:** Nutritional assessment before and following the 4-week supplementation period.

	Placebo		SidTea+^TM^	
Variable	Pre	Post	Pre	Post
Energy (Kcal/day)	1741 ± 541	1775 ± 600	1682 ± 381	1743 ± 379
Carbohydrates (g/day)	180.1 ± 76.3	189.3 ± 76.3	160.1 ± 59.8	164.3 ± 60.8
Carbohydrates (% of total energy/day)	40.6 ± 9.4	42.2 ± 9.6	37.7 ± 10.7	38 ± 12.2
Fat (g/day)	86.5 ± 33.7	85.3 ± 31.5	81.4 ± 26.9	83.2 ± 28.8
Fat (% of total energy/day)	43.6 ± 10.2	42.5 ± 10.1	42.6 ± 9.7	42.2 ± 10.1
Protein (g/day)	64.3 ± 29.1	64.0 ± 27.8	68.9 ± 25.9	69.9 ± 26.5
Protein (% of total energy/day)	14.5 ± 4.4	14.4 ± 4.3	16.2 ± 5.0	15.9 ± 4.8
Cholesterol (mg/day)	323 ± 304	317 ± 289	339 ± 296	337 ± 278
Selenium (μg/day)	115.1 ± 91.0	116 ± 90.8	123.0 ± 87.8	123.6 ± 87.3
Vitamin A (IU/day)	2407 ± 2121	2527 ± 2164	2617 ± 2368	2507 ± 2775
Vitamin C (mg/day)	88.2 ± 57.5	89.4 ± 58.3	85.2 ± 61.9	86.3 ± 62.9
Vitamin E (mg/day)	7.0 ± 4.6	7.9 ± 4.9	6.8 ± 4.3	7.5 ± 4.6

**Table 3:** Assessment of anthropometric, physiological and performance characteristics before and following the 4-week supplementation period.

	Placebo		SidTea+^TM^	
Variable	Pre	Post	Pre	Post
Body mass (kg)	83.5 ± 19.4	83.83 ± 19.3	72.9 ± 15.9	72.7 ± 15.3
Body height (m)	1.71 ± 0.1	1.71 ± 0.1	1.69 ± 0.1	1.69 ± 0.1
BMI (kg/m^2^)	28.3 ± 5.7	28.4 ± 5.7	25.2 ± 4.0	25.2 ± 3.9
Waist circumference (cm)	93.9 ± 15.3	92.0 ± 15.9	85.5 ± 11.2	82.7 ± 12.7
Hip circumference (cm)	106.2 ± 10.4	106.8 ± 10.0	100.9 ± 8.3	100.6 ± 7
Waist/Hip (ratio)	0.88 ± 0.1	0.86 ± 0.1	0.85 ± 0.1	0.82 ± 0.1
Body fat (%)	31.4 ± 9.1	30.2 ± 9.1	28.1 ± 6.6	27.7 ± 6.6
Fat mass (kg)	27.1 ± 11.9	26.0 ± 11.6	20.6 ± 7.1	20.3 ± 7.0
Fat-free mass (kg)	56.5 ± 11.6	57.8 ± 12.4	52.3 ± 12.3	52.5 ± 11.9
Systolic BP (mm Hg)	121.0 ± 15.4	115.1 ± 12.1	118.8 ± 15.5	107.9 ± 8.6 **
Diastolic BP (mm Hg)	73.9 ± 8.1	76.7 ± 9.2	72.9 ± 6.7	71.5 ± 8.7
Mean arterial pressure (mm Hg)	89.6 ± 9.3	89.5 ± 9.7	88.2 ± 8.6	83.7 ± 7.8 *
Resting heart rate (b/min)	63.6 ± 8.4	61.8 ± 9.2	64.2 ± 12.8	61.1 ± 10.1 *
Handgrip strength-DL (kg)	38.2 ± 12.4	32.4 ± 11.7 **	30.4 ± 11.3	29.6 ± 9.6
Handgrip strength-NDL (kg)	32.4 ± 12.2	29.0 ± 9.8 *	28.6 ± 9.6	25.8 ± 8.0 *
eVO_2_max (mL/kg/min)	43.0 ± 8.8	42.7 ± 8.7	40.5 ± 7.2	41.6 ± 5.9 *

* Significant within-group difference with the pre value (*p* < 0.05). ** Significant within-group difference with the pre-value (*p* < 0.01). BMI, body mass index; BP, blood pressure; DL, dominant limb; NDL, non-dominant limb; eVO_2_max, estimated maximal oxygen uptake.

**Table 4:** Complete blood count assessment before and following the 4-week supplementation period.

	Placebo		SidTea+^TM^	
Variable	Pre	Post	Pre	Post
White blood cells (10^3^/μL)	6.6 ± 1.2	6.7 ± 1.7	6.2 ± 1.9	6.4 ± 1.6
Lymphocytes (10^3^/μL)	2.4 ± 0.5	2.6 ± 1.1	2.2 ± 0.8	2.3 ± 0.7
Monocytes (10^3^/μL)	0.3 ± 0.1	0.4 ± 0.1	0.3 ± 0.1	0.3 ± 0.1
Granulocytes (10^3^/μL)	3.9 ± 0.9	3.8 ± 1.1	3.7 ± 1.7	3.7 ± 1.0
Lymphocytes (%)	36.4 ± 5.1	38.0 ± 9.0	36.2 ± 9.0	36.6 ± 6.1
Monocytes (%)	4.8 ± 0.8	5.4 ± 1.1	4.9 ± 2.1	5.1 ± 1.0
Granulocytes (%)	58.7 ± 4.6	56.6 ± 9.1	58.8 ± 10.1	58.3 ± 6.7
Red blood cells (10^6^/μL)	5.1 ± 0.3	4.9 ± 0.3	5.0 ± 0.4	4.9 ± 0.3
Hemoglobin (g/dL)	14.2 ± 1.1	13.9 ± 1.4	13.5 ± 1.8	13.2 ± 1.7
Hematocrit (%)	43.5 ± 2.1	42.4 ± 3.4	42.0 ± 3.9	41.6 ± 4.1
MCV (μm^3^)	86.3 ± 5.6	86.8 ± 6.2	85.1 ± 7.3	85.0 ± 7.2
MCH (pg)	28.2 ± 2.4	28.5 ± 2.6	27.4 ± 3.4	27.0 ± 3.0
MCHC (g/dL)	32.6 ± 1.2	32.8 ± 1.0	32.1 ± 1.5	31.7 ± 1.1
RDW (%)	14.7 ± 0.8	14.5 ± 1.0	14.8 ± 1.2	14.7 ± 1.4
PLT (10^3^/μL)	254.9 ± 34.5	232.4 ± 29.0	239.7 ± 34.3	234.2 ± 46.0
MPV (μm^3^)	7.7 ± 0.6	7.8 ± 0.5	8.0 ± 0.5	8.0 ± 0.3
PCT (%)	0.2 ± 0.0	0.2 ± 0.0	0.2 ± 0.0	0.2 ± 0.0
PDW (%)	15.6 ± 1.4	15.2 ± 1.9	15.7 ± 1.0	15.4 ± 0.7

MCV, mean corpuscular volume; MCH, mean corpuscular hemoglobin; MCHC, mean corpuscular hemoglobin concentration; RDW, red blood cell distribution width; PLT, platelets; MPV, mean platelet volume; PCT, volume occupied by platelets in the blood as a percentage; PDW, platelet distribution width.

**Table 5:** Assessment of metabolic markers before and following the 4-week supplementation period.

	Placebo		SidTea+^TM^	
Variable	Pre	Post	Pre	Post
Glucose (mg/dL)	88.0 ± 10.4	89.67 ± 10.5	87.7 ± 10.7	85.8 ± 8.5
Cholesterol (mg/dL)	206.4 ± 28.9	199.6 ± 32.9	199.0 ± 37.3	195.5 ± 44.9
HDL (mg/dL)	51.3 ± 12.8	51.1 ± 14.4	48.9 ± 7.1	48.5 ± 9.3
LDL (mg/dL)	138.4 ± 26.6	132.9 ± 29.9	131.7 ± 31.1	129.7 ± 36.7
Triglycerides (mg/dL)	83.6 ± 31.9	78.00 ± 30.5	92.1 ± 48.6	86.4 ± 44.8
Bilirubin (mg/dL)	1.03 ± 0.61	1.00 ± 0.74	0.76 ± 0.84	0.91 ± 1.26
γ-GT (U/L)	27.9 ± 14.2	27.9 ± 14.3	25.2 ± 17.4	21.5 ± 12.1 *
SGOT (U/L)	20.89 ± 4.8	20.2 ± 5.5	20.2 ± 5.7	19.4 ± 6.1
SGPT (U/L)	18.6 ± 7.7	19.9 ± 10.3	19.8 ± 8.3	16.5 ± 6.4 *
Creatinine (mg/dL)	1.11 ± 0.20	1.12 ± 0.22	0.97 ± 0.13	0.96 ± 0.11
Uric acid (mg/dL)	5.59 ± 1.7	5.59 ± 1.9	5.17 ± 1.5	4.95 ± 1.3
LDH (U/L)	311.4 ± 64.9	297.8 ± 50.9	290.1 ± 52.8	295.8 ± 47.4

* Significant within-group difference with the pre-value (*p* < 0.05). HDL, high-density lipoprotein; LDL, low-density lipoprotein; γ-GT, gamma-glutamyl transpeptidase; SGOT, serum glutamic-oxaloacetic transaminase; SGPT, serum glutamic-pyruvic transaminase; LDH, lactate dehydrogenase.

## Data Availability

Data supporting the findings of the present study are available from the corresponding author upon reasonable request.

## Supplementary Materials

The following supporting information can be downloaded at: https://www.mdpi.com/article/10.3390/molecules29051113/s1, Table S1: CONSORT 2010 checklist of information to include when reporting a randomized trial [62].
